# Genotype–phenotype correlation of β-lactamase-producing uropathogenic *Escherichia coli* (UPEC) strains from Bangladesh

**DOI:** 10.1038/s41598-020-71213-5

**Published:** 2020-09-03

**Authors:** Maqsud Hossain, Tahmina Tabassum, Aura Rahman, Arman Hossain, Tamanna Afroze, Abdul Mueed Ibne Momen, Abdus Sadique, Mrinmoy Sarker, Fariza Shams, Ahmed Ishtiaque, Abdul Khaleque, Munirul Alam, Anwar Huq, Gias U. Ahsan, Rita R. Colwell

**Affiliations:** 1 NSU Genome Research Institute (NGRI), North South University, Dhaka, Bangladesh; 2 Department of Biochemistry and Microbiology, North South University, Dhaka, Bangladesh; 3 International Centre for Diarrheal Disease Research, Bangladesh (icddr,b), Dhaka, Bangladesh; 4 Maryland Pathogen Research Institute, University of Maryland, College Park, MD USA; 5 Department of Public Health, North South University, Dhaka, Bangladesh; 6 University of Maryland Institute of Advanced Computer Studies, University of Maryland, College Park, MD USA; 7 Johns Hopkins Bloomberg School of Public Health, Baltimore, MD USA

**Keywords:** Infectious-disease diagnostics, Pathogens

## Abstract

*Escherichia coli* is a pathogen commonly encountered in clinical laboratories, and is capable of causing a variety of diseases, both within the intestinal tract (intestinal pathogenic strains) and outside (extraintestinal pathogenic *E. coli*, or ExPEC). It is associated with urinary tract infections (UTIs), one of the most common infectious diseases in the world. This report represents the first comparative analysis of the draft genome sequences of 11 uropathogenic *E. coli* (UPEC) strains isolated from two tertiary hospitals located in Dhaka and Sylhet, Bangladesh, and is focused on comparing their genomic characteristics to each other and to other available UPEC strains. Multilocus sequence typing (MLST) confirmed the strains belong to ST59, ST131, ST219, ST361, ST410, ST448 and ST4204, with one of the isolates classified as a previously undocumented ST. De novo identification of the antibiotic resistance genes *bla*_NDM-5_, *bla*_NDM-7_, *bla*_CTX-M-15_ and *bla*_OXA-1_ was determined, and phenotypic-genotypic analysis of virulence revealed significant heterogeneity within UPEC phylogroups.

## Introduction

Urinary tract infections (UTIs) are the most common bacterial infections affecting approximately 11% of adult women each year globally, with approximately 60% of women experiencing UTI during their lifetime^1, 2^. Sporadic studies done on the prevalence of UTIs in Bangladesh and an investigation of 200 UTI patients, including men and women of various age groups, found females to be more susceptible to UTIs (80% positive) than males. In both genders, the prevalence rate was highest among those in the age group of 21–40 years (33%)^3^. The study also showed *E. coli* to be the predominant etiological agent, contributing to 57.38% of infections^3^.

*Escherichia coli* is an extremely diverse bacterial species which can be categorized into three major groups based on disease causing capability: commensal or nonpathogenic *E*. *coli*; intestinal pathogenic *E*. *coli* causing diarrhea; and extraintestinal pathogenic *E*. *coli* (ExPEC). The ExPEC term was described by Johnson et al. in 2000^4^ and further subclassified as uropathogenic *E. coli* (UPEC), sepsis-associated *E*. *coli* (SEPEC), and neonatal meningitis-associated *E. coli* (MNEC)^5^. ExPECs are known to invade extraintestinal tissue and cause pathogenesis by harboring a variety of virulence factors, either present in the chromosome or carried in mobile genetic elements such as plasmids, thereby conferring greater diversity among ExPEC strains^5,6^.

Traditionally, *E. coli* phylogroups B2 and D have been understood to cause the majority of ExPEC infections, while phylogroups A and B1 were associated with comensal extraintestinal strains^7^. However, recent reports have revealed higher percentages of phylogroup A strains in UTI cases^8^. A strong association has also often been detected between a particular multilocus sequence type (MLST) with a pathology, such as the correlation of globally dominant *E. coli* ST131 and extraintestinal infections, especially in India^9^. Like ST131, many other successful clonal lineages of different sequence types (ST), including 410, 95 and 10 have disseminated globally due to their relatively higher virulence, fitness, and metabolic capabilities, along with acquisition of antibiotic resistance genes^10–13^.

Carbapenems are considered last-resort antibiotics, therefore resistance to this group of antibiotics is a greater health concern in treating infections caused by extended-spectrum-β-lactamase (ESBL)- or AmpC-producing bacteria^14^. New Delhi metallo-β-lactamase (NDM) is a relatively recent group of metallo-β-lactamase (MBL) that, over the last decade, has undergone rapid spread in the South-Asian continent^15^. While NDM producers have been found to be susceptible to a few antibiotics including colistin, several recent studies have reported that this treatment approach might not be sustainable and could become a very serious public health concern^16,17^. NDM genes are found both in plasmids and chromosomally integrated in various bacterial pathogens^18,19^. Reports of *E. coli* harboring NDM-1 and other ESBL genes such as CTX-M and OXA-48 have emerged from various parts of the world, including Japan, the Netherlands, South Korea, and Tanzania^10–12,20–23^. In addition, many such studies have detected the chromosomal integration of NDM genes in various ExPEC sequence types, such as ST38, ST410, ST131 and ST648^24^.

Association of different virulence factors, e.g., *sat* (secreted autotransporter toxin), *iutA* (aerobactin (siderophore) receptor), *malX* (pathogenicity island marker) and *ompT* (outer-membrane protease T), has also been reported, with specific sequence types such as ST38, ST131, ST405, and ST648 isolated^25^. In general, however, few genomic investigations have been done that could shed light on molecular mechanisms of pathogenesis and antibiotic resistance mechanisms and correlate those traits with the genotypes of local pathogens, especially in a developing country like Bangladesh.

To the best of our knowledge, there is no genomic information available on UPEC isolates circulating in Bangladesh. This study represents an initial effort to obtain genomic information on Bangladeshi UPEC isolates and to analyze genomic variations between Bangladeshi isolates and ExPECs from different parts of the world. Eleven strains representing different ExPEC phylogroups and antibiotic resistance were selected and their genomes determined using next-generation sequencing. Genotype–phenotype correlation analyses were also done on the isolates to determine virulence properties, e.g., biofilm formation, serum resistance, hemolysis, and antibiotic resistance.

## Results

### Antibiogram and phylogroup analysis

Presumptive identification using colony morphology revealed 47 of 74 (63.5%) bacterial isolates obtained from the Dhaka Central International Medical College and Hospital (DCIMCH) and 19 of 32 isolates (59.4%) from the Ibn Sina Hospital, Sylhet (ISH) were *E. coli*. All isolates from DCIMCH exhibited increased resistance to commonly used antimicrobials, including β-lactams (third- and fourth-generation cephalosporins), fluoroquinolones, and aminoglycosides (Table 1; Supplementary Table S1). Antibiotic sensitivity patterns differed between isolates collected from the two hospitals, with isolates from DCIMCH showing resistance to a larger number of antibiotics. For example, while most of the DCIMCH isolates were resistant to cefixime (85.1%), only 26.3% from ISH showed resistance to this antibiotic. DCIMCH isolates showed high frequency of resistance to second generation cephalosporin cefuroxime (83%), third generation cephalosporins ceftriaxone (80.9%) and ciprofloxacin (72.3%), and the monobactam, aztreonam (72.3%). ISH isolates, in contrast, showed resistance mainly to doxycycline (42.1%) and amoxicillin (36.8%). While *ca*. 17% (n = 8) of DCIMCH isolates conferred resistance to the carbapenem, imipenem, all of the ISH isolates were sensitive to carbapenems and all isolates included in this study were sensitive to colistin. Five isolates from DCIMCH were ESBL (Extended Spectrum β-lactamase) positive, but none from ISH were positive (Table S1).

**Table 1:** Percentage of DCIMCH and ISH strains resistant to different antibiotics.

DCIMCH		ISH	
n = 47 [female = 25, male = 22]		n = 19 [female = 10, male = 9]	
Colistin	0%	Colistin	0%
Polymixin B	0%	Polymixin B	0%
Cefotaxime	80.90%	Ceftriaxone	5.26%
Ceftriaxone	80.90%	Ceftazidime	0%
Ceftazidime	46.80%	Cefixime	26.30%
Cefixime	85.10%	Cefoxitin	26.32%
Cefuroxime	80.90%	Imipenem	0%
Cefepime	55.30%	Meropenem	0%
Aztreonam	72.30%	Doxycycline	42.10%
Imipenem	17.00%	Gentamicin	0%
Meropenem	19.10%	Mecillinam	31.60%
Ciprofloxacin	72.30%	Amoxicillin	36.80%
Gentamicin	25.50%	Azithromycin	31.60%
Co-trimoxazole	51.10%	Trimethoprim	26.32%
Levofloxacin	63.80%		
Nalidixic Acid	80.90%		
Netilmicin	12.80%		
Nitrofurantoin	17.02%		
Piperacillin/Tazobactum	34.00%		
Tigecycline	2.13%		
Amikacin	14.90%		
Amoxyclave	55.30%		

Phylogroup determination based on PCR detection of *chuA*, *yjaA* and TspE4.C2^26^, showed phylogroup B2 and phylogroup A to be most abundant, with B2 comprising 19 (40.4%) and A 14 (29.8%) of the 47 isolates (Table S2). A total of 11 strains (23.4%) were classified in phylogroup B1, while three (6.4%) were phylogroup D. Seven of 66 isolates harboured NDM-1 gene and all NDM positive strains were from DCIMCH. No association was observed between NDM and a particular phylogroup, with three strains from phylogroup A, two strains from B1, and one strain from B2 carrying the NDM gene.

### Genomic features and strain characterization

Eleven isolates from the phylogroups were selected with the number of isolates from each phylogroup roughly proportional to prevalence of that phylogroup within the set of 47 isolates in this study. These were selected based on resistance patterns. Four isolates were selected from phylogroups A and B2, two from phylogroup B1 (NGE5 and NGCE100), and one from phylogroup D (NGE3). Combined length of contigs of the assembled genomes of each of the 11 strains ranged from ~ 4.3 to 5.4 Mbp, with N50 value (the minimum contig length required to cover 50% of the genome) ranging between 58,987 and 363,834 bp (Table 2). Size of the pangenome (i.e. total gene repertoire) was 16,797 genes and core genome 2,945 genes.

**Table 2:** Genome assembly statistics of the 11 sequenced UPEC isolates.

Sample name	Strain ID	Accession number	Genome size	Number of contigs (> 500 bp)	Largest contig size	N50 value
NGRI_A12	NGE3	QEXN00000000	5,104,547	147	319,826	167,222
NGRI_A13	NGE4	QFAZ00000000	4,786,247	105	339,704	206,487
NGRI_A14	NGE5	RCIF00000000	4,669,166	54	1,088,366	354,566
NGRI_A15	NGE6	RCIE00000000	4,885,404	59	711,364	363,834
NGRI_A16	NGE7	QFRN00000000	5,304,720	101	655,033	204,841
NGRI_A18	NGE9	QFRT00000000	4,254,362	205	190,686	58,987
NGRI_B10	NGE16	QFTM00000000	5,232,178	305	179,874	60,840
NGRI_B29	NGE22	QFXA00000000	5,061,598	134	338,320	157,136
NGRI_C17	NGCE33	RBWA00000000	4,805,154	99	553,030	204,552
NGRI_C19	NGCE94	RAZR00000000	5,143,790	204	262,871	108,692
NGRI_C20	NGCE100	RAZQ00000000	5,422,176	244	313,308	105,294

De novo analysis was used to confirm phylogroups of the assembled genomes and MLST analysis showed that, while strains belonging to a phylogroup were heterogenous in MLST types, there was direct correlation between serotype and MLST, with ST131 strains NGE7 and NGE16 both serotype O25:H4 (Table 3).

**Table 3:** De novo prediction of phylogroups, MLST types and serotypes of the sequenced UPEC isolates.

Strains	Hospital	Phylogroups	MLST type	Serotype
NGE3	DCIMCH	D	ST-59	O1:H7
NGE4	DCIMCH	A	ST-4204	O6:H10
NGE5	DCIMCH	B1	Unknown ST	O59:H20
NGE6	DCIMCH	B2	ST-219	O138:H48
NGE7	DCIMCH	B2	ST-131	O25:H4
NGE9	DCIMCH	B2	ST-219	O138:H48
NGE16	ISH	B2	ST-131	O25:H4
NGE22	ISH	A	ST-4204	O6:H10
NGCE33	DCIMCH	A	ST-410	O8:H9
NGCE94	DCIMCH	A	ST-361	O9:H30
NGCE100	DCIMCH	B1	ST-448	Ounknown:H7

### Phylogenetic and cluster dendrogram analysis

The 11 strains were compared to obtain the number of SNPs shared between any two strains. From the SNP matrix shown in Supplementary Table S3, isolates with the same ST shared a low SNP count, while isolates within the same phylogroup but different STs had high SNPs. For example, NGE7, NGE16, and reference strain (NA114) belong to ST131 and share a low SNP count of 411 bp, whereas ST219 strains (NGE9 and NGE6) and ST131 strains had high SNP count but were the same phylogroup.

**Figure 1:**
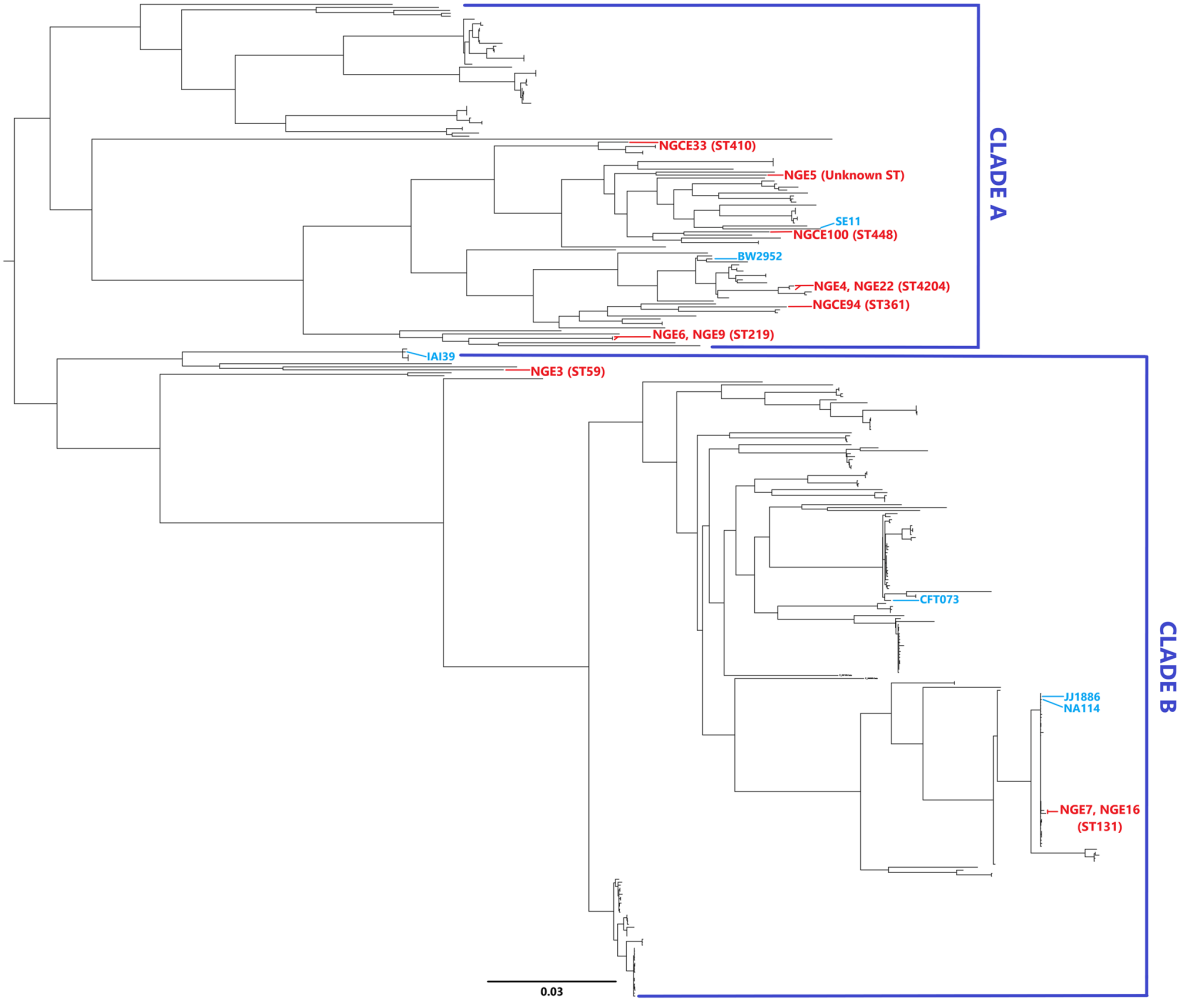
Phylogenomic organization of publicly accessible UPEC isolates with sequenced Bangladeshi isolates in this study. Mid-point rooted SNPtree demonstrates the phylogenetic distribution of 11 UPEC genomes of Bangladeshi UPEC isolates amongst 5 UPEC reference genomes and 386 UPEC genomes (isolated from both urine and blood) available online. The well characterized reference genomes and UPEC isolates of this study have been labeled in blue and red respectively.

Core alignment using parSNP aligned 189 of 402 UPEC strains available online (list of strains is given in Supplementary Table S4). A total of the 60,815 SNPs identified was extracted and linked to construct a midpoint rooted phylogenetic tree (Fig. 1), showing two major clades, Clade A and Clade B. Clade A branched into subclades, with strains from phylogroup A and B1 in one subclade and ST219 strain of phylogroup B2 in another. NGCE33 is an ESBL-containing, highly virulent strain of ST410 which, despite belonging to phylogroup A, clustered distantly from the rest of phylogroup A strains (NGE22, NGE4 and NGCE94) and closer to phylogroup B1 (NGE5, NGCE100 and SE11). Clade B also branched phylogroup D and B2 away from each other. It was observed that isolates obtained from urine and blood samples interleaved, without significant clustering of infection type. However, NGE5 (proposed as a new ST) in clade A, NGE7, and NGE16 (ST131) in clade B joined strains isolated from blood. Strains belonging to the same MLST were placed together in the phylogenetic tree.

**Figure 2:**
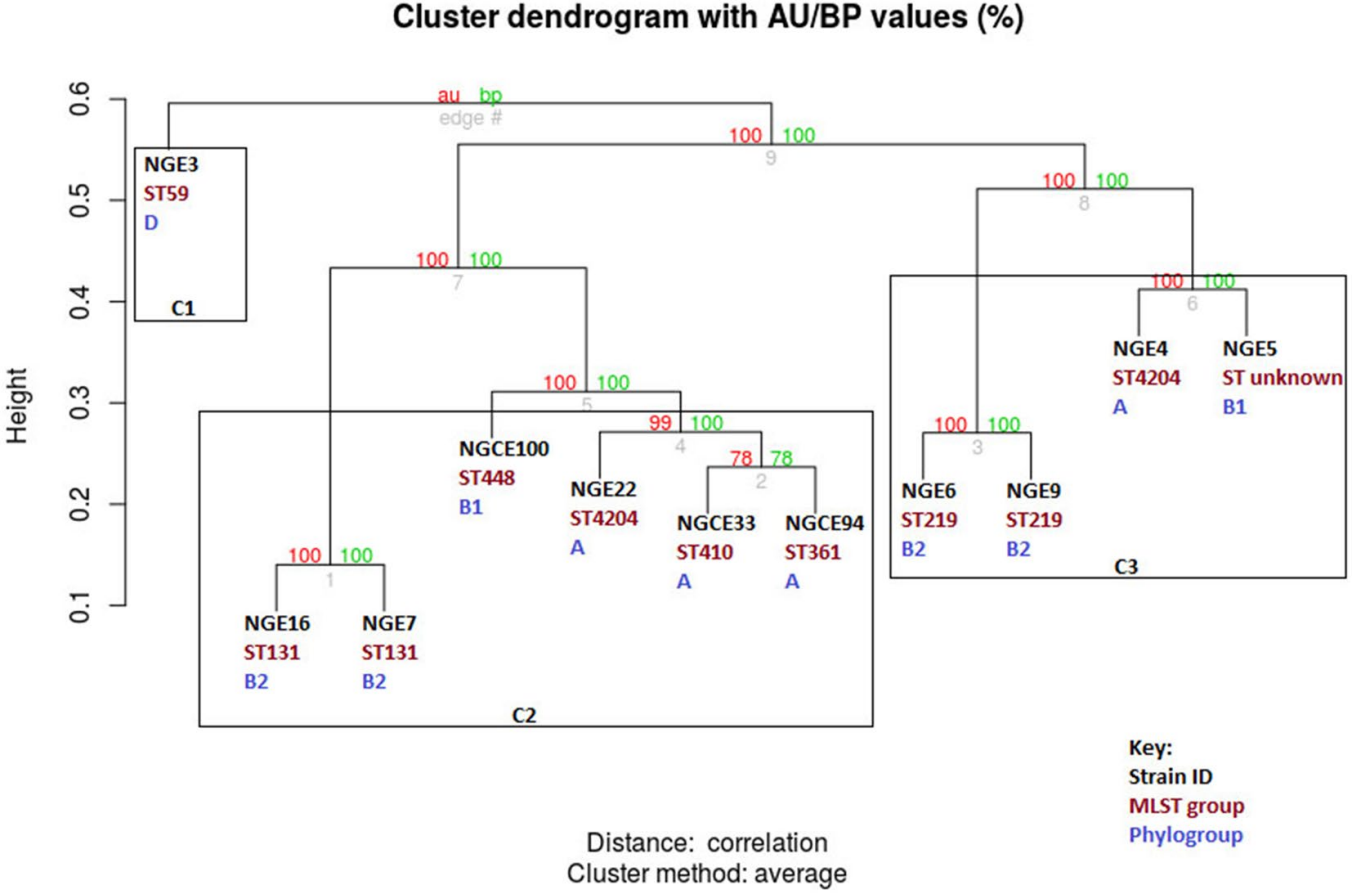
Dendrogram illustrating pan genome clustering of sequenced UPEC isolates. Dendrogram branches into three major clusters (C1, C2 and C3) based on the presence and absence of characterized accessory genes in the pan genome.

Hierarchical clustering of the 11 UPEC strain sequences represents similarity of shared accessory genome content, yielding three major clusters, C1, C2, and C3, respectively (Fig. 2). Phylogroup D out grouped, forming a distinct cluster, C1, while a combination of phylogroups A, B1, and B2 joined the remaining two clusters (C2 and C3). C2 further divided into two distinguishable clusters comprising strains of ST131 family in one group and four multidrug resistant, highly virulent strains of various MLSTs in another. These four isolates included one ST448 strain, one ST361 strain carrying both *bla*_NDM_ and ESBL genes, one “high-risk” clone of ST410 lineage^27^, and one resistant strain of the ST4204 family. The clustering pattern of C2 suggests sharing of accessory genes between highly virulent strains, irrespective of phylogroups and ST. C3 also separated into two clusters, comprising two less virulent phylogroup B2 strains of the ST219 family in one clade, and the moderately virulent ST4204 strain of phylogroup A (NGE4) and the phylogroup B1 strain of unknown ST (NGE5) in the other.

Accessory gene distribution responsible for this hierarchical clustering pattern is listed in Supplementary Table S5. Strains in cluster C2 share genes including OriC-binding nucleoid-associated protein (*cnu*), hemolysin expression-modulating protein (*hha*)*,* suppressor of T4 td mutant (*stpA*) responsible for regulation of hemolysin (*hly*) gene expression and genes such as periplasmic inhibitor of g-type lysozyme (*pliG*) which provides lysozyme tolerance. Similarly, shared genomic content in cluster C3 involves genes *RenD, ybcN* and *ybcK* coding for uncharacterized prophage related proteins absent in C1 and C2 strains. Strains ST131 and ST219 belong to phylogroup B2 yet are located in two different clusters as ST131 strains contain 223 unique genes with significant enrichment in genes involved in biosynthetic and metabolic processes absent in ST219 strains, thus explaining the high numbers of SNPs between strains of the two STs.

### De novo identification of antibiotic resistance markers

**Figure 3:**
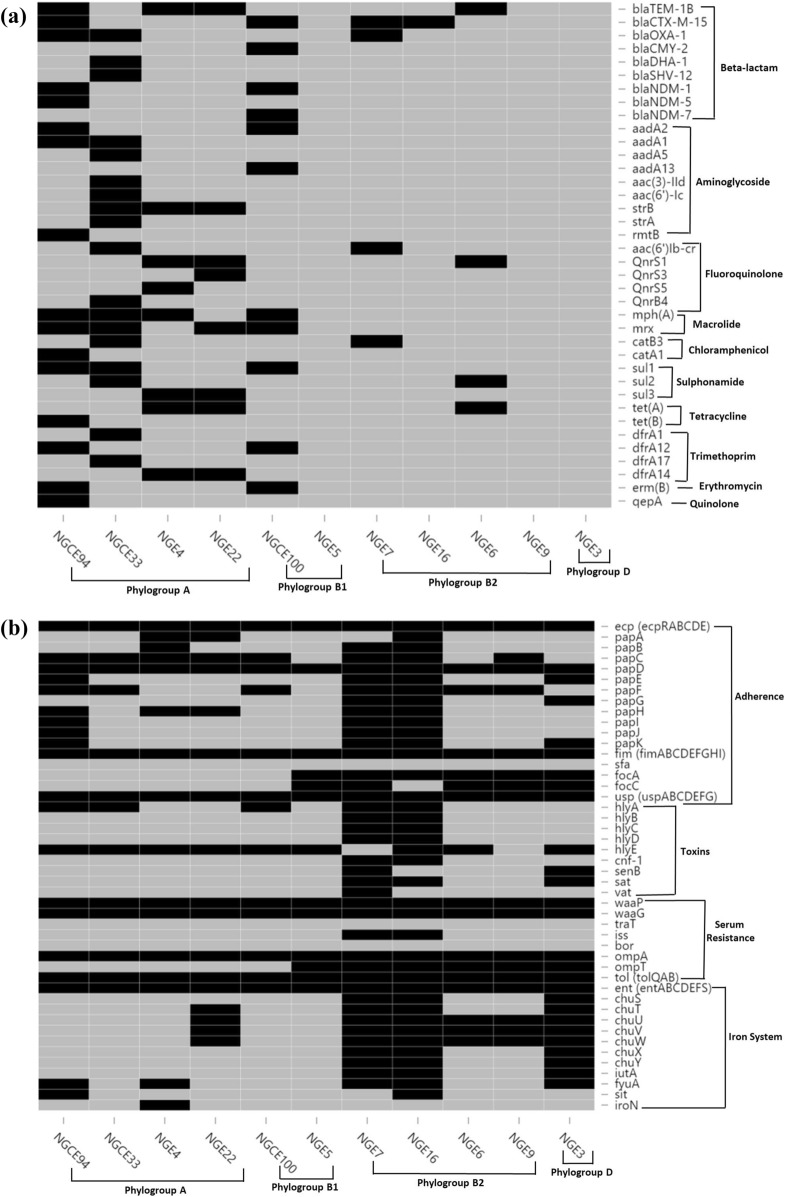
(**a**) Distribution of key antimicrobial resistance conferring genes within the 11 strains. (**b**) Distribution of key virulence factors within the 11 strains. Black: present, grey: absent.

Genome annotation revealed that chromosomes of all strains sequenced had previously been reported to carry intrinsic antibiotic resistance genes, such as *marA, gyrA, parC* and *parE*^28^, as well as plasmid-mediated resistance genes belonging to AMR families, including β-lactamases, fluoroquinolones, aminoglycosides, macrolides, tetracyclines, trimethoprims, and sulfonamides. Genes associated with antibiotic resistance are shown in Fig. 3a.

Eleven isolates, irrespective of phylogroup and sequence type (ST), showed resistance to antibiotics according to presence of resistance genes. ESBL genotype *bla*_CTX-M-15_ is a predominant gene present in two NDM positive strains (NGCE100, NGCE94) and two ST131 strains (NGE16 and NGE7). NDM positive strains possessing *bla*_NDM-5_ and *bla*_NDM-7_ belong to phylogroup A and B1, and MLST groups ST361 and ST448, respectively. In addition, NGCE94 (ST361) contained β-lactamases *bla*_TEM-1B_ and *bla*_OXA-1_, tetracycline-resistance *tet*(*B*), quinolone-resistance gene *qepA*, trimethoprim-resistance gene *dfrA12* and chloramphenicol-resistance gene *catA1*. Variants of *bla*_TEM_ and *dfrA* were also present in the highly resistant ST4204 strains (NGE4 and NGE22) belonging to the same phylogroup as ST361. Relatively less resistant ST219 strains (NGE6 and NGE9) of phylogroup B2 present a similar resistance pattern and contain resistance markers (*tet*(A) and *qnrS1*) common to phylogroup A rather than phylogroup B. Another member of phylogroup A (NGCE33) included highly resistant ST410 containing an array of β-lactamase genes including *bla*_OXA-1B_*, bla*_CMY-2_*, bla*_DHA-1_ and *bla*_SHV-12_. In addition, it contained a number of aminoglycosidase genes, fluoroquinolone-resistance gene *qnrB4*, macrolide gene *mph*(A) and trimethoprim-resistance genes *drfA1* and *drfA17*. Genes *strB* and *mph*(A) were also shared by ST4204 isolates. Moreover, genes *rmtB, aadA1, mph*(A) and erythromycin-resistance gene *erm*(B) were harboured by NDM positive strains. Only a few resistance genes such as *acc*(*6′*)*lb-cr, catB3* and *bla*_OXA-1_ were shared between the ST131 and ST410 strains.

Overall, the most resistant strain was NGCE33, based on genes coding for β-lactamase resistance, including the ESBL, bla_CTX-M-15_. This strain and NGCE100 were resistant to nitrofurantoin, a last resort nephrotoxic antibiotic that recently is more commonly used to treat carbapenem resistant UTIs.

### Analysis of virulence profiles and genotype–phenotype correlation

UPEC pathogenesis encompasses a range of mechanisms including colonization of the urinary tract, protection against host defenses, and toxin production. Hemolysin production of 11 strains was tested, using blood agar and, while alpha hemolytic activity was observed only for ST131 strains NGE7 and NGE16, mild hemolysis was detected for NGE3, NGE4, and NGE22.

**Figure 4:**
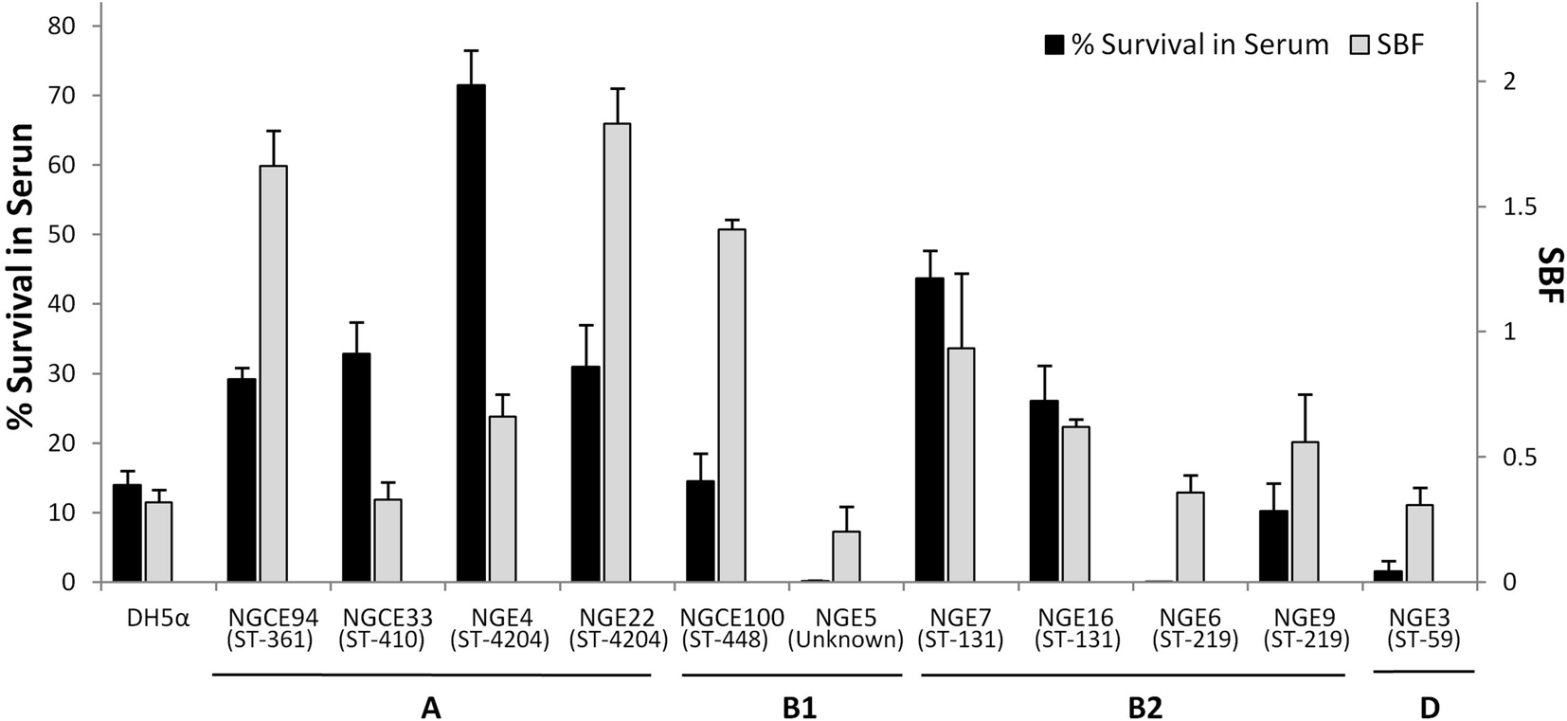
Serum resistance and biofilm forming propensities of 11 *E. coli* isolates. Pooled NHS was inoculated with overnight LB culture in 1:10 ratio. Bacteria were enumerated at 0 and 3 h of incubation at 37 °C, and percentage survival in serum was calculated. For biofilm assay, bacteria were grown in M63 media and specific biofilm formation (SBF) was calculated. Error bars represent standard error (SE).

An important pathogenic determinant of UPEC is ability to form biofilm^29^. The biofilm formation assay results showed variation in biofilm formation both between and within the phylogroups (Fig. 4). Three strains (NGE22, NGCE94 and NGCE100) were classified as strong biofilm formers and four (NGE4, NGE7, NGE9 and NGE16) were moderate biofilm formers, based on specific biofilm formation value (SBF). Differences in biofilm forming ability were observed by sequence type, as exemplified by NGE7 and NGE16, both in the pandemic ST131 family, as well as ST219 strains (NGE6 and NGE9) and ST4204 strains (NGE4 and NGE22).

Bactericidal activity of serum complement acts as a major first-line defense against bacterial infection infiltrated into tissue^30^. In vitro serum resistance assay results showed all strains were susceptible to human serum bactericidal activity to varying degrees (Fig. 3). Except for NGE3, NGE5, and NGE6, they showed survival capacity equal to or greater than that of DH5α. While serum has a more pronounced bactericidal activity for strains belonging to phylogroup B1 and D, most strains of phylogroups A and B2 exhibited better survival in serum. Although little correlation was observed between degree of biofilm formation and serum resistance (r = 0.188) (Fig. 4), moderate/strong biofilm formers all showed greater survival in serum compared to weak biofilm formers.

Pan-genome analyses revealed virulomes of the 11 strains (Fig. 3b). In general, ST131 strains NGE7 and NGE16 carry an extensive repertoire of virulence genes. While the core genome encompasses gene families from different classes of virulence factors, not all are conserved, according to the SNP/bp ratio (Table 4).

**Table 4:** SNP distribution of core virulence genes.

Gene	Gene function	SNP count	Gene size (bp)	Alignment size (bp)	SNP/bp
*ompA*	Outer Membrane Protein A	59	1,054	1,054	0.056
*uspA*	Universal Stress Protein A	79	438	435	0.181
*uspB*	Universal Stress Protein B	4	336	336	0.012
*uspC*	Universal Stress Protein C	12	430	430	0.028
*uspD*	Universal Stress Protein D	11	429	429	0.026
*uspE*	Universal Stress Protein E	29	951	951	0.03
*uspF*	Universal Stress Protein F	7	435	435	0.016
*uspG*	Universal Stress Protein G	34	429	429	0.079
*tolA*	Tol-Pal System Protein A	33	1,311	1,311	0.025
*tolB*	Tol-Pal System Protein B	30	1,293	1,293	0.023
*tolQ*	Tol-Pal System Protein Q	29	693	693	0.042
*entA*	2,3-Dihydro-2,3-dihydroxybenzoate dehydrogenase	50	747	747	0.067
*entB*	Enterobactin Synthase Component B	37	858	858	0.043
*entC*	Isochorismate Synthase	58	1,176	1,176	0.049
*entD*	Enterobactin Synthase Component D	121	783	660	0.183
*entE*	Enterobactin Synthase Component E	116	1,611	1,611	0.072
*entF*	Enterobactin Synthase Component F	322	3,882	3,882	0.083
*entS*	Enterobactin Synthase Component S	117	1,251	1,251	0.094
*fimA*	Type 1 fimbral protein, chain A	218	567	567	0.384
*fimC*	Chaperone Protein	23	726	726	0.032
*fimD*	Outer Membrane Usher Protein	89	2,637	2,637	0.034
*fimF*	Type 1 fimbral protein, chain F	23	531	531	0.043
*fimG*	Type 1 fimbral protein, chain G	12	504	504	0.024
*fimH*	Type 1 fimbrin d-mannose specific adhesin	34	903	903	0.038

Comparison with VFDB indicated adhesion factors belonging to the *ecp* (*Escherichia coli* common pilus)*, csg* (Curli fibers) and *fim* (type 1 fimbriae) gene families are part of the core genetic pool, while *afaB/C* (adherence fibrillar adhesion) and *sfa* (S fimbrial adhesion) are absent. Variation was noted in presence of the *foc* (F1C fimbriae) and *pap* (pyelonephritis associated pili) family genes. SNP distributions show that while other members of the *fim* genes are moderately conserved, *fimA* displays high genetic variability with SNP/bp of 0.383. Among the conserved *usp* (universal stress protein) genes that are involved in bacterial adhesion, *uspA* also indicated greater variation with SNP/bp of 0.181. Among toxicity conferring genes, hemolysin toxin *hlyA* was detected in five strains, including the two ST131 strains. Other genes, such as *hlyB, hlyC*, *hlyD, cnf-1* and *sat,* were present exclusively in only ST131 strains.

All 11 strains carried the well-characterized serum resistance gene *ompA*, while *traT* and *bor* were missing from their genomes. Only ST131 strains harbored *iss* (increased serum survival) another important gene in the serum resistome of *E. coli*. The genomes were also analyzed for presence of 56 genes recently characterized as belonging to the serum resistome of EC985 by transposon-directed insertion site sequencing (TraDIS)^31^. Most of these genes, including *tol(A,B,Q)* and *rfaH* were detected in the core genome. However, some essential genes were either completely absent (*hyxA* and *hyxR*) or not identical to the reference strain from the VFDB database (*waaP* and *waaG*).

### Analysis of pathogenicity islands (PAIs)

CFT073, a well characterized pyelonephritogenic strain, harbours PAIs that suggest strong virulence when present in UPEC isolates^32,33^. Many UTI associated strains, as well as commensal *E. coli*, carry PAIs that were first identified in strain 536^34^. To determine genetic composition of PAIs, genomes of the 11 UPEC isolates were analyzed for presence of the CFT073 and 536 associated PAI genes as retrieved from the PAthogenisity Island DataBase (https://www.paidb.re.kr/) (Supplementary Table S6).

**Figure 5:**
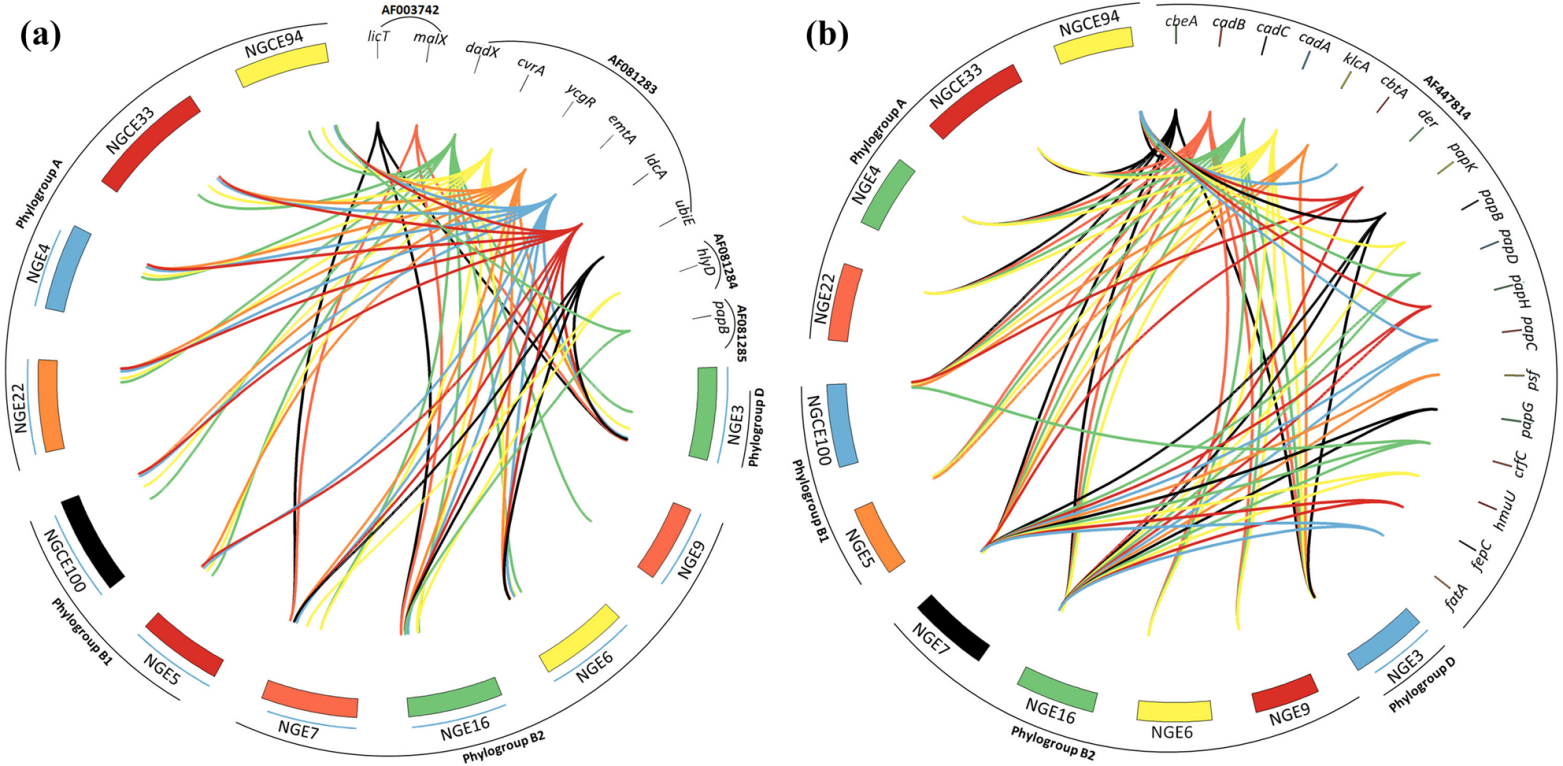
Distribution of genes associated with a. PAI I_CFT073_ and b. PAI II_CFT073_ in 11 UPEC genomes. Relational diagrams were generated using CIRCOS software. Bands were created for each gene of CFT073 PAI-I and PAI-II along with the 11 sequenced strains to depict the presence of CFT073 associated PAI genes in the strains.

Analysis of PAI I_CFT073_ (Fig. 5a) showed that only two ST131 strains (NGE7 and NGE16) and NGE3 carried *malX*, a phosphotransferase system enzyme coding gene linked with occurrence of extraintestinal infections. The PAI I_CFT073_ gene *dadX*, however, was detected in all strains, while other genes, such as those coding for the motility regulating factors *ycgR* and *emtA*, a peptidogycan recycling enzyme *ldcA* (L, D-carboxypeptidase A) and *cvrA* (putative K^+^:H^+^ antiporter), were detected in all strains except NGE9. Apart from *cad *(*BAC*) which was present in all strains, most of the PAI II_CFT073_ associated genes (Fig. 5b) were found only in the ST131 isolates, NGE7 and NGE16. However, a phylogroup A strain NGCE94, was found to carry several genes of the *pap* (P fimbriae coding) operon indicating the possibility of acquisition of PAI II_CFT073_ from more virulent strains in the genitourinary microenvironment.

Analysis of Strain 536 associated genetic elements showed that PAI I_536_ (containing hemolysin genes *hlyA, B, C,* and *D*) as well as PAI II_536_ were exclusively found in NGE7 and NGE16. PAI III_536_ gene *yciC* (UPF0259 membrane protein) was only present in NGE3, NGE7 and NGE16. Furthermore, all strains apart from NGE3, NGE4 and NGE22 were enriched with genes from PAI V_536_. PAI IV_536_ genes on the contrary, had more diffuse distribution among the strains with only *mtfA* (mnemonic for Mlc titration factor A). That PAI IV_536_ is considered more stable than other PAIs of strain 536^35^ may explain these results.

## Discussion

High-throughput sequencing technology development has led to a significant decrease in the cost of whole genome sequencing of bacterial pathogens so that sequencing is routine in developed countries. Infrastructure for next generation sequencing is now being developed in research centers located in developing countries including Bangladesh. The NSU Genome Research Institute (NGRI) at North South University, Bangladesh, has been established and aims to decipher whole genomes of bacterial pathogens of public health concern to Bangladesh. This initial study was carried out to determine genome characteristics of UPEC strains circulating in the country by sequencing 11 UPEC strains representing prevalent ExPEC phylogroups and antibiotic resistance profiles.

ST410 has been previously detected in Southeast Europe, Middle East and Greece^28^ and classified as a “high-risk clone” with essential regular monitoring due to its enhanced resistance mechanisms and moderate virulence^36^. The resistance and virulence patterns of ST410 strain NGCE33 validates this finding, and its very first identification in Bangladesh emphasizes the extent of its spread across continents. This study also reports an unknown MLST type i.e. NGE5, an observation that highlights the rapidly mutating nature of the UPEC genome and indicates the value of genomic characterization of local isolates.

The phylogenetic tree based on difference in SNPs (Fig. 1) showed phylogroups A and B1 branching separately from phylogroups B2 and D. This finding supports previous reports of phylogroup A and B1 belonging to sister lineages^36,37^. Phylogroup D and B2 can be concluded to have the same ancestral origin since most of the strains of these phylogroups are located in the same clade (Clade B) in SNP tree. A lack of clear phylogenomic separation of strains isolated from urine and blood coincides with results of previous related work^28,38^. The difference in SNP counts observed in this study further elucidates genomic relatedness since these sites account for the variation in nucleotide sequence. It was observed that ST219 and ST131 strains have an increased number of SNP difference despite belonging to the same phylogroup and thus cluster in separate clades in the phylogenetic tree. This may be due to difference in pathogenic capabilities and resistance potentials between STs, i.e., gain and loss of accessory genome in genomic diversity, with strains harbouring different fitness factors and MDR genotypes, irrespective of ST and phylogroup. Comparative genomic analysis of the sequenced UPEC strains showed that the phylogenetic analysis is congruous with serotype, sequence type, virulence and AMR pattern.

This is further supported by results of the genomic and phenotypic analyses (Fig. 4). The genetic architectures of strains included in this study are concordant with previous findings, with well known pandemic strains such as ST131 possessing many virulence genes and enriched PAIs^9^. However, as supported by the results of this study, although ST131 strains exhibit a notably dense virulome compared to other sequenced strains, a stark difference was not observed between the phenotypic virulence of ST131 and non ST131 strains (Fig. 4). Strains within the same phylogroup also displayed varied levels of virulence. While ST131 strains of phylogroup B2 (NGE7 and NGE16) displayed relatively strong phenotypic virulence, another strain NGE6, also classified under phylogroup B2 but of ST219 origin, displayed a much weaker pathogenic potential, such as weak biofilm formation.

Closer scrutiny of virulomes of the sequenced strains reveal certain virulence factors pertaining to resistance against serum bactericidal activity were either completely missing or present in degraded form within the bacterial genomes. These discrepancies explain that while most strains are capable of enduring serum bactericidal activity, they do not possess the robust serum resistome required to overcome complement action and proliferate in serum. The lack of any clear association between a particular gene and a given virulence phenotype suggests that likely there is a combinatorial effect of genes on pathogenic potential. *E. coli* possesses an open pan-genome by continuing gene acquisition, as found in other studies^38,39^. This characteristic may also explain phenotypic results as mentioned above and acquisition of yet-to-be characterized new genes may determine pathogenicity of strains. Several recent studies report an altered pathogenic potential of commensal *E. coli*^40^ and a similar observation can be made from this study. Emergence of highly virulent strains belonging to phylogroup A may be due to the open pan genome nature of *E. coli*, allowing it to acquire new virulence factors and resistance markers. SNP analyses of core virulence factors reveal that certain genes within a given gene family, such as *fimA,* are prominently more polymorphic compared with other members.

Several findings have indicated that septicemic/pyelonephritogenic strains carry certain virulent genes located in mobile genetic elements, called pathogenicity islands (PAI), usually absent in avirulent or less virulent strains^41^. Of all the strains sequenced in this study, ST131 strains NGE7 and NGE16 predictably possessed the most gene-dense CFT073 and 536 associated PAIs (Fig. 5). However, the presence of a large number of PAI II_CFT073_ mediated adhesion factors in the genome of NGCE94 indicates dissemination of virulence traits via horizontal transfer of PAIs from commonly virulent phylogroup B2 strains to less virulent phylogroup A strains. Transfer and evolution of genetic elements like PAIs contribute to fitness and pathogenic properties of UPEC.

Rapid emergence of multidrug resistant (MDR) strains of *E. coli* is a very serious concern, especially in a developing country like Bangladesh which experiences antibiotic misuse. Most resistance properties emerge via intra-species horizontal resistance gene transfer^42^. Clearly, however, excessive use of antibiotics in the global community creates evolutionary pressure towards enhanced resistance of UPEC^28^. Reported spread of the pan beta-lactam antibiotic resistance *bla*_NDM_ family of genes is a major cause for concern, because of resistance conferred against penems, cephamycins, cephalosporins, and carbapenems, as well as horizontal transfer since the gene is located in a plasmid. This study reveals the presence of NDM gene varieties and a number of other ESBL genes in phylogroup A and B1 strains. In addition, the spread of virulence properties to strains like NGCE94, which is both NDM and ESBL positive, can have a profound effect on healthcare and spread of disease in Bangladesh.

In conclusion, this study presents useful insight into the genomes of Bangladeshi UPEC isolates, notably reporting for the first time an emerging pandemic clone ST410 in Bangladesh, contributing to the global distribution of this lineage. The study also demonstrates that strains belonging to phylogroup A exhibit virulence characteristics comparable to globally predominant known virulent ST131 (phylogroup B2) isolates, while other phylogroup B2 strains, such as ST219, display lower pathogenic potential. It also substantiates classification based on sequence type being an improved measure of genomic relatedness and pathogenicity. The risks posed by emerging pathogenic strains within different phylogroups need further assessment using comparative genomics and larger sample size.

## Methods

### Selection of isolates and antibiotic resistance profiling

A total of 106 bacterial isolates were cultured from urine of patients suffering from UTI and admitted to either the intensive care or emergency unit of two tertiary hospitals in Bangladesh from the period of June, 2017 and July, 2018. A total of 74 isolates were from inpatients admitted to Dhaka Central International Medical College and Hospital (DCIMCH), Dhaka and 32 isolates were collected from inpatients at Ibn Sina Hospital, Sylhet (ISH). *E. coli* colonies were presumptively identified by their colony morphology on MacConkey agar. Antibiotic susceptibility was carried out using the disc diffusion method and included 22 antibiotics for the 47 strains isolated from DCIMCH and 16 antibiotics for the 19 ISH strains (Table S1). Results were interpreted according to the 27th edition of Clinical and Laboratory Standards Institute (CLSI) guidelines. The isolates were transferred to North South University Genome Research Institute (NGRI), Dhaka, Bangladesh for further analysis.

### PCR amplification and gel electrophoresis

The 66 *E. coli* isolates were inoculated into LB broth (HiMedia, India), grown at 37 °C overnight, and DNA was extracted using GeneJET Genomic DNA Purification Kit (Cat. No K0721) (ThermoFisher Scientific, USA) according to manufacturer’s protocol. Conventional singleplex PCR was carried out to detect the NDM gene^43^, and to classify strains into phylogroups using previously described protocols (Table S2)^26^. In brief, 12.5 µl of reaction volume was used containing 6.25 µl DreamTaq Green PCR Master Mix (ThermoFisher Scientific, USA), 1.0 µl 25 nmol of MgCl_2_, 20 pmol of forward and reverse primers and *ca.* 100 ng DNA. Amplification was carried out using GeneAtlas (Astec Co, Ltd), with the following assay conditions: denaturation at 94 °C for 5 min; 30 cycles of 30 s at 94 °C, 30 s at annealing temperature, 30 s at 72 °C, and final extension at 72 °C for 5 min. Agarose gel electrophoresis was used to visualize banding patterns of the strains.

### Genome assembly and annotation

Library preparation and sequencing of the 11 selected strains were conducted at NGRI. *Ca.* 1 μg of high molecular weight *E. coli* genomic DNA was used to prepare Illumina libraries and employing Nextera DNA Library Preparation Kit (Cat. No. FC-121-1030) according to manufacturer’s guideline. De novo assembly of good quality paired-end Illumina reads (Q ≥ 30) was done by running genome assembly software SPAdes (v3.12)^44^ with filters to decrease the number of mismatches and short indels. Assembled contigs were annotated using PROKKA pipeline^45^ with contiglength < 500 bp filtered out. Possible genomic contaminations were assessed using the ContEst16S tool^46^. Pan and core genome size of the 11 isolates and reference genome NA114^47^ were identified using the GF (Gene Family) method of pan-genome analysis pipeline (PGAP) (v1.2.1)^48^. Further pan and core genome analyses were performed using Roary^49^. Hierarchical clustering based on presence and absence of accessory genes, was performed using PVclust: R package^50^, based on bootstrap resampling to generate *p-*values. The bootstrap value was set to n = 1,000, to cross-validate the clustering pattern. The functions of the accessory genes were analysed via STRING database (https://string-db.org/).

### Evolutionary relationship and phylogenomic analysis

Single Nucleotide Polymorphism (SNP) matrix was generated using CSIphylogeny 1.4 (Conserved Signature Indels) (Table S3)^51^. The 11 sequenced UPEC isolates from this study, 386 publicly available published genomes^12,38^, and a few reference strains of diverse STs and phylogroups, including NA114^47^, CFT073^52^, IAI39^53^, SE11^54^ and BW2952^55^ (Table S4), were aligned to generate a core alignment in order to derive whole genome SNP using Parsnp v1.2 from the Harvest suite^56^. SNP file was processed by SNPRelate: R package^57^ and phylogenomic tree was visualized using FigTree (https://tree.bio.ed.ac.uk/software/figtree/).

### In silico analysis of UPEC genome sequences

Phylogroups were confirmed based on presence of marker genes *arpA, chuA, yjaA* and TspE4.C2^26^ using local BLAST the scheme set by Clermont et al*.* in 2012^58^. The ST of each annotated genome was extracted from the MLST 2.0 (Multi-locus sequence typing) database^59^, and serotypes were determined using SerotypeFinder 2.0^60^. Similarly, antimicrobial resistance (AMR) genes and plasmids of each isolate was obtained from ResFinder 3.1^61^ and PlasmidFinder 2.0^62^ respectively. Furthermore, a virulence factor profile was generated by amalgamation of results obtained using BLASTp against the Virulence Factors of Bacterial Pathogens database (VFDB)^63^ that had been made available in 2016, and the tool VirulenceFinder 2.0^51^. SNPs per gene were calculated using DnaSP v6^64^. To study the genetic composition of possible PAIs, genomes of the 11 UPEC isolates were analysed for presence of CFT073 and 536 associated PAI genes retrieved from the PAthogenisity Island DataBase (https://www.paidb.re.kr/)^65^ and visualized using CIRCOS^66^.

### Phenotypic virulence determination

Alpha and beta haemolytic reactions of the strains were demonstrated using blood agar (Oxoid, UK), prepared using sheep blood. A single isolated colony for each strain was streaked on a blood agar plate which was incubated overnight at 37 °C*.* Partially clear and completely clear zones around the colony were indicative of alpha and beta hemolytic activity respectively.

Biofilm formation assays were performed using previously described protocols with minor modifications^13^. Bacteria were grown overnight in M63 at 37 °C after which 2 µl aliquots were added to 198 µl fresh M63 medium in a sterile 96-well polystyrene microtitre plate with four replicate wells for each strain. M63 broth without inoculum served as negative control. The plates were incubated statically at 37 °C for 24 h and OD_600_ was measured both at the beginning of incubation (0 h) and end of incubation at 24 h (GloMax, Promega). The culture was then discarded and plates were gently washed twice with sterile saline and air dried. *Ca.* 250 µl 0.1% crystal violet was added to the wells and allowed to stain for 15 min. The plates were then washed thrice with distilled water and air dried. The stained bacterial cells were resolubilized in 200 µl of 33% glacial acetic acid and the plates were read at 560 nm to enumerate cells in the biofilm. Specific biofilm formation (SBF) was measured using the formula SBF = (AB – CW)/G, where AB is OD_560_ of stained cells, CW is OD_560_ of control wells, and G is bacterial cell growth calculated using the formula G = OD_600nm(24 h)_ – OD_600nm(0 h)_. The strains were classified as follows: SBF < 0.5 = weak biofilm former, 0.5 ≤ SBF < 1.0 = moderate biofilm former and SBF ≥ 1.0 = strong biofilm former^67^. The entire assay was performed at least twice for each strain.

Assay for serum resistance was performed using a slightly modified version of previously described protocols^13^. *Ca.* 5 µl from overnight cultures was added to 495 µl fresh LB broth (HiMedia, India) and inoculated statically for 2 h at 37 °C. The culture was then centrifuged at 5,000×*g* for 7 min and the pellet obtained was suspended in 500 µl of sterile saline. *Ca.* 20 µl aliquots from this mixture were transferred to 180 µl of normal human serum (NHS) in a sterile 96-well microtitre plate and incubated at 37 °C under static conditions for 3 h. *Ca.* 20 µl was removed from the culture at 0 h and after 3 h incubation and plated on LB plates after serial dilution. Bacteria were enumerated after the plates had incubated overnight at 37 °C. Resistance to serum was measured as percentage change in colony forming units (CFU) at the beginning and end of the incubation period. The entire experiment was run in duplicate.

### Ethical statements

All isolates included in this study were collected for diagnostic purposes from two local tertiary hospitals where pathogens are isolated from clinical specimens as part of a routine diagnostic procedure and not for experimental purposes. All experiments and methods were carried out in accordance with relevant guidelines and regulations. All experimental protocols were approved by the North South University (NSU) Institutional Review Board (IRB) / Ethical Review Committee (ERC), protocol No. CTRG:NSU-RP-18-042. Clinical isolates used in this study were recovered for diagnostic purposes from local diagnostic centers or hospitals and were not experimental in nature. The clinical data were anonymized and unlinked and the requirement for informed consent was waived by the NSU IRB/ERC.

## Supplementary information

Supplementary Tables.

## Data Availability

The 11 uropathogenic *E. coli* genome sequences and analysis from this study have been submitted to GenBank database, with accession numbers that include QEXN00000000 (NGE3), QFAZ00000000 (NGE4), RCIF00000000 (NGE5), RCIE00000000 (NGE6), QFRN00000000 (NGE7), QFRT00000000 (NGE9), QFTM00000000 (NGE16), QFXA00000000 (NGE22), RBWA00000000 (NGCE33), RAZR00000000 (NGCE94) and RAZQ00000000 (NGCE100).
