# Supplementary material for: Genotype–phenotype correlation of β-lactamase-producing uropathogenic Escherichia coli (UPEC) strains from Bangladesh
Source: Sci Rep. 2020 Sep 3;10:14549. doi: 10.1038/s41598-020-71213-5 (PMC7471317; doi:10.1038/s41598-020-71213-5)
Supplement: Supplementary file 1 — Supplementary Tables. [file 41598_2020_71213_MOESM1_ESM.pdf]

Genotype-phenotype correlation of β-Lactamase-producing Uropathogenic *Escherichia coli* (UPEC) strains from Bangladesh

Maqsud Hossain[a,b], Tahmina Tabassum[a,b]†, Aura Rahman[a,b]†, Arman Hossain[a,b], Tamanna Afroze[a], Abdul Mueed Ibne Momen[a,b], Abdus Sadique[a], Mrinmoy Sarker[b], Fariza Shams[b], Ahmed Ishtiaque[b], Abdul Khaleque[b], Munirul Alam[c], Anwar Huq[d], Gias U Ahsan[a,e], Rita R Colwell[d,f,g#]

†Equal contribution

[a] NSU Genome Research Institute (NGRI), North South University, Dhaka, Bangladesh

[b] Department of Biochemistry and Microbiology, North South University, Dhaka, Bangladesh

[c] International Centre for Diarrheal Disease Research, Bangladesh (ICDDR,B), Dhaka, Bangladesh

[d] Maryland Pathogen Research Institute, University of Maryland, College Park, Maryland, USA

[e] Department of Public Health, North South University, Dhaka, Bangladesh

[f] University of Maryland Institute of Advanced Computer Studies, University of Maryland, College Park, Maryland, USA

[g] Johns Hopkins Bloomberg School of Public Health, Baltimore, Maryland, USA

Supplementary Table S1: Antibiogram results and ESBL status of 66 UPEC isolates. The sequenced strains are highlighted in red. R= Resistant; S= Susceptible; I= Intermediate.

DHAKA HOSPITAL ID	STRAIN ID	GENDER	AGE	SAMPLE TYPE	ESBL	Amikacin	Amoxyclave	Aztreonam	Cefepime	Cefixime	Cefotaxime	Ceftazidime	Ceftriaxone	Cefuroxime	Ciprofloxacin	Colistin	Co-trimoxazole	Gentamicin	Imipenem	Levofloxacin	Meropenem	Nalidixic Acid	Netilmicin	Nitrofurantoin	Piperacillin/ Tazobactum	Polymixin B	Tigecycline
77827	NGCE-002	MALE	28	URINE	-	S	R	S	R	R	R	S	R	R	R	S	R	R	S	R	S	R	S	R	R	S	S
78463	NGCE-003	FEMALE	39	URINE	-	S	S	S	S	S	S	S	S	S	R	S	S	S	S	S	S	R	S	S	S	S	S
78773	NGCE-004	MALE	64	URINE	-	S	R	S	S	S	S	S	S	S	R	S	S	S	S	R	S	R	S	S	S	S	S
77937	NGCE-005	FEMALE	18	URINE	-	S	R	S	R	R	R	S	S	S	S	S	S	S	S	S	S	R	S	S	S	S	S
79078	NGCE-006	MALE	24	URINE	-	S	S	S	S	S	S	S	S	S	S	S	S	S	S	S	S	S	S	S	S	S	S
78156	NGCE-007	FEMALE	71	URINE	-	S	R	S	R	R	R	S	R	R	R	S	S	S	S	R	S	R	S	S	S	S	S
80245	NGCE-009	MALE	35	URINE	-	S	R	S	S	S	S	S	S	S	S	S	S	S	S	S	S	S	S	S	S	S	S
100001	NGCE-032	MALE	37	URINE	-	S	S	R	S	R	R	S	R	R	R	S	R	S	S	R	S	R	S	S	S	S	S
200002	NGCE-033	FEMALE	1	URINE	-	R	R	R	R	R	R	R	R	R	R	S	R	R	R	R	R	R	R	R	R	S	S
300003	NGCE-034	MALE	1	URINE	-	R	R	R	R	R	R	R	R	R	R	S	R	R	R	R	S	R	R	R	R	S	S
400004	NGCE-035	FEMALE	59	URINE	-	R	R	R	R	R	R	S	R	R	R	S	R	R	R	R	S	R	R	R	R	S	R
152802	NGCE-056	FEMALE	50	URINE	-	S	S	R	S	R	S	S	R	R	S	S	S	S	S	S	S	R	S	S	S	S	S
152875	NGCE-061	FEMALE	45	URINE	-	S	S	R	R	R	R	R	R	R	R	S	S	S	S	R	S	R	S	S	S	S	S
155231	NGCE-065	FEMALE	72	URINE	-	S	S	R	S	R	R	R	R	R	S	S	S	R	S	S	R	R	S	S	S	S	S
155237	NGCE-066	MALE	26	URINE	-	S	R	R	R	R	R	R	R	R	S	S	R	S	S	S	S	S	S	S	R	S	S
24780	NGCE-068	FEMALE	6	URINE	-	S	R	R	R	R	R	R	R	R	R	S	S	S	S	S	R	R	S	S	S	S	S
K-1122	NGCE-069	MALE	7	URINE	-	S	R	S	S	R	S	S	S	R	R	S	R	S	S	R	R	S	S	S	S	S	S
25269	NGCE-079	MALE	69	URINE	-	S	R	R	R	R	R	R	R	R	R	S	R	S	S	R	S	R	S	S	S	S	S
157224	NGCE-080	FEMALE	60	URINE	-	S	S	R	S	R	R	S	R	R	R	S	S	S	S	R	S	R	S	S	S	S	S
25259	NGCE-084	FEMALE	60	URINE	-	S	R	R	R	R	R	R	R	R	R	S	S	S	S	R	S	R	S	R	R	S	S
158532	NGCE-094	MALE	63	URINE	+	R	R	R	R	R	R	R	R	R	R	S	R	R	R	R	R	R	R	S	R	S	S
160790	NGCE-100	FEMALE	18	URINE	+	S	R	R	R	R	R	R	R	R	R	S	R	S	R	R	R	R	S	R	R	S	S
160010	NGCE-101	MALE	63	URINE	-	R	R	R	R	R	R	R	R	R	R	S	R	R	R	R	R	R	R	S	R	S	S
160937	NGCE-102	MALE	2	URINE	-	S	R	R	R	R	R	S	R	R	R	S	R	R	S	R	S	R	S	S	S	S	S
160826	NGCE-103	FEMALE	16	URINE	-	S	S	R	S	R	R	S	R	R	S	S	S	S	S	S	S	S	S	S	S	S	S
160139	NGCE-112	MALE	0	URINE	-	S	R	R	R	R	R	R	R	R	R	S	R	R	S	R	S	R	S	S	R	S	S
161487	NGCE-118	FEMALE	68	URINE	-	S	R	R	R	R	R	R	R	R	R	S	R	S	S	R	S	R	S	S	R	S	S
26385	NGCE-137	MALE	68	URINE	+	S	S	R	R	R	R	R	R	R	S	S	S	S	S	S	S	S	S	S	S	S	S
162300	NGCE-139	MALE	68	URINE	+	S	R	R	R	R	R	R	R	R	R	S	R	S	R	R	R	R	S	S	R	S	S
164238	NGCE-148	FEMALE	30	URINE	+	S	R	R	R	R	R	R	R	R	R	S	R	S	S	R	S	R	S	R	S	S	S
165890	NGCE-155	FEMALE	1	URINE	-	S	S	R	S	R	R	S	R	R	S	S	S	S	S	S	S	S	S	S	S	S	S
165185	NGCE-159	MALE	58	URINE	-	S	S	R	S	R	R	S	R	R	R	S	R	S	S	R	S	R	S	S	S	S	S
169361	NGCE-166	MALE	72	URINE	-	S	R	R	R	R	R	R	R	R	R	S	R	S	S	S	S	R	S	S	R	S	S
169703	NGCE-171	FEMALE	50	URINE	-	S	S	R	S	R	R	S	R	R	R	S	S	S	S	R	S	R	S	S	S	S	S
169692	NGCE-173	MALE	4	URINE	-	S	S	R	S	R	R	S	R	R	R	S	S	S	S	R	S	R	S	S	S	S	S
27375	NGCE-174	FEMALE	47	URINE	-	S	R	R	R	R	R	R	R	R	R	S	S	S	S	R	S	R	S	R	R	S	S
184208	NGCE-177	MALE	78	URINE	-	R	R	R	S	R	R	S	R	R	R	S	R	S	S	R	S	R	S	S	S	S	S
170119	NGCE-179	MALE	86	URINE	-	S	S	R	S	R	R	S	R	R	R	S	R	S	S	R	S	R	S	S	S	S	S
171218	NGCE-182	FEMALE	45	URINE	-	S	R	R	R	R	R	R	R	R	S	S	S	S	S	S	S	S	S	S	R	S	S
171517	NGCE-187	FEMALE	24	URINE	-	S	R	R	R	R	R	R	R	R	R	S	S	R	S	R	S	R	S	S	S	S	S
173094	NGCE-203	MALE	65	URINE	-	S	S	S	S	S	S	S	S	S	R	S	R	R	S	R	S	R	S	S	S	S	S
30823	NGCE-205	FEMALE	60	URINE	-	S	S	S	S	S	S	S	S	S	S	S	S	S	S	S	S	R	S	S	S	S	S
180140	NGCE-213	FEMALE	1	URINE	-	S	S	S	S	R	R	S	R	R	R	S	R	S	S	R	S	R	S	S	S	S	S
180174	NGCE-214	FEMALE	48	URINE	-	S	S	S	S	R	R	S	R	R	R	S	R	S	S	S	S	R	S	S	S	S	S
182401	NGCE-218	FEMALE	55	URINE	-	S	S	R	R	R	R	R	R	R	S	S	S	S	S	S	S	S	S	S	S	S	S
182448	NGCE-219	FEMALE	49	URINE	-	S	S	S	S	S	S	S	S	S	S	S	S	S	S	S	S	R	S	S	S	S	S
183940	NGCE-225	MALE	56	URINE	-	R	R	R	R	R	R	R	R	R	R	S	R	R	R	R	R	R	R	S	R	S	S

SYLHET HOSPITAL ID	STRAIN ID	GENDER	AGE	SAMPLE TYPE	ESBL	Amoxycillin	Azithromycin	Cefixime	Ceftazidime	Ceftriaxone	Colistin	Doxycycline	Gentamicin	Imipenem	Mecillinam	Meropenem	Polymixin B	Trimethoprim	Cefoxitin
88185	NGCE-010	FEMALE	47	URINE	-	S	R	R	S	R	S	R	S	S	R	S	S	R	R
147975	NGCE-012	MALE	73	URINE	-	S	S	S	S	S	S	S	S	S	S	S	S	S	S
147661	NGCE-013	FEMALE	19	URINE	-	S	S	S	S	S	S	S	S	S	S	S	S	S	S
146156	NGCE-014	MALE	34	URINE	-	S	S	S	S	S	S	S	S	S	S	S	S	S	S
146605	NGCE-015	FEMALE	44	URINE	-	S	S	S	S	S	S	S	S	S	S	S	S	S	S
147639	NGCE-016	MALE	36	URINE	-	R	S	R	S	S	S	S	S	S	R	S	S	S	R
Q-020	NGCE-019	FEMALE	23	URINE	-	S	S	S	S	S	S	S	S	S	S	S	S	S	S
Q-27118	NGCE-020	MALE	41	URINE	-	R	R	R	S	S	S	R	S	S	R	S	S	R	R
Q-27183	NGCE-021	FEMALE	65	URINE	-	S	S	S	S	S	S	S	S	S	S	S	S	S	S
Q-260	NGCE-022	MALE	68	URINE	-	R	R	S	S	S	S	R	S	S	R	S	S	R	S
Q-149714	NGCE-023	FEMALE	32	URINE	-	S	S	S	S	S	S	S	S	S	S	S	S	S	S
Q-458	NGCE-024	MALE	81	URINE	-	R	R	S	S	S	S	R	S	S	S	S	S	R	R
Q-149777	NGCE-025	FEMALE	17	URINE	-	S	S	S	S	S	S	S	S	S	S	S	S	S	S
Q-084	NGCE-026	MALE	12	URINE	-	R	R	R	S	S	S	R	S	S	S	S	S	R	R
Q-149517	NGCE-027	FEMALE	3	URINE	-	S	S	S	S	S	S	S	S	S	S	S	S	S	S
Q-14	NGCE-028	MALE	45	URINE	-	S	S	S	S	S	S	S	S	S	S	S	S	S	S
Q-295	NGCE-029	FEMALE	2	URINE	-	R	S	S	S	S	S	R	S	S	R	S	S	S	S
Q-903	NGCE-030	MALE	42	URINE	-	S	S	S	S	S	S	R	S	S	S	S	S	S	S
Q-079	NGCE-031	FEMALE	33	URINE	-	R	R	R	S	S	S	R	S	S	R	S	S	R	R

Supplementary Table S2: The distribution of phylogroup determining genes as detected by PCR. Sequenced strains are highlighted in red.

Strain ID	*chuA*	*yjaA*	TspE4.C2	Phylogroup	NDM
NGCE2	na	na	na	na	-
NGE3	+	-	-	D	-
NGE4	-	+	-	A	-
NGE5	-	-	+	B1	-
NGE6	+	+	-	B2	-
NGE7	+	+	+	B2	-
NGE9	+	+	-	B2	-
NGCE10	-	-	+	B1	-
NGCE12	na	na	na	na	-
NGCE13	na	na	na	na	-
NGCE14	-	-	-	A	-
NGCE15	+	+	+	B2	-
NGE16	+	+	+	B2	-
NGCE19	-	+	+	B1	-
NGCE20	+	+	+	B2	-
NGCE21	-	+	+	B1	-
NGE22	-	+	-	A	-
NGCE23	-	+	-	A	-
NGCE24	-	+	-	A	-
NGCE25	+	+	+	B2	-
NGCE26	-	-	-	A	-
NGCE27	na	na	na	na	-
NGCE28	-	-	-	A	-
NGCE29	-	+	-	A	-
NGCE30	-	-	-	A	-
NGCE31	+	+	+	B2	-
NGCE32	+	+	-	B2	-
NGCE33	-	+	-	A	+
NGCE34	na	na	na	na	+
NGCE35	-	+	-	A	+
NGCE56	na	na	na	na	-
NGCE61	na	na	na	na	-
NGCE65	na	na	na	na	-
NGCE66	na	na	na	na	-
NGCE68	+	+	+	B2	-
NGCE69	+	+	+	B2	-
NGCE79	-	-	+	B1	-
NGCE80	na	na	na	na	-
NGCE84	na	na	na	na	-
NGCE94	-	+	-	A	+
NGCE100	-	-	+	B1	+
NGCE101	+	+	-	B2	+
NGCE102	+	+	+	B2	-
NGCE103	-	-	-	A	-
NGCE112	na	na	na	na	-
NGCE118	na	na	na	na	-
NGCE137	+	+	-	B2	-
NGCE139	-	+	+	B1	-
NGCE148	-	+	-	A	-
NGCE155	-	-	+	B1	+
NGCE159	na	na	na	na	-
NGCE166	na	na	na	na	-
NGCE171	-	+	+	B1	-
NGCE173	-	+	+	B1	-
NGCE174	+	-	+	D	-
NGCE177	na	na	na	na	-
NGCE179	na	na	na	na	-
NGCE182	+	+	+	B2	-
NGCE187	na	na	na	na	-
NGCE203	-	-	+	B1	-
NGCE205	+	-	+	D	-
NGCE213	+	+	+	B2	-
NGCE214	+	+	+	B2	-
NGCE218	+	+	+	B2	-
NGCE219	+	+	+	B2	-
NGCE225	na	na	na	na	-

Supplementary Table S3: Matrix showing the variation in SNP counts between the sequenced UPEC isolates.

Strains	NGCE100	NGCE33	NGCE94	NGE16	NGE22	NGE3	NGE4	NGE5	NGE6	NGE7	NGE9	Ref (NA114)
NGCE100	0	16608	21432	35478	22848	39181	22784	13432	29469	35510	29454	35303
NGCE33	16608	0	21102	34271	22818	39033	22757	17095	29559	34307	29567	34089
NGCE94	21432	21102	0	35725	16856	39545	16800	22280	28792	35756	28785	35548
NGE16	35478	34271	35725	0	35797	30928	35749	35761	35332	411	35342	331
NGE22	22848	22818	16856	35797	0	38690	324	24045	29790	35831	29791	35622
NGE3	39181	39033	39545	30928	38690	0	38641	39201	38935	30954	38960	30777
NGE4	22784	22757	16800	35749	324	38641	0	23975	29728	35783	29734	35576
NGE5	13432	17095	22280	35761	24045	39201	23975	0	29513	35789	29485	35590
NGE6	29469	29559	28792	35332	29790	38935	29728	29513	0	35363	274	35150
NGE7	35510	34307	35756	411	35831	30954	35783	35789	35363	0	35375	370
NGE9	29454	29567	28785	35342	29791	38960	29734	29485	274	35375	0	35158
Ref (NA114)	35303	34089	35548	331	35622	30777	35576	35590	35150	370	35158	0

Supplementary Table S4: List of the genomes used in the phylogenomic tree construction and analysis

Source	Strains
This study	NGE3, NGE4, NGE5, NGE6, NGE7, NGE9, NGE16, NGE22, NGCE33, NGCE94, NGCE100
Andersen *et al.* 2013	Escherichia coli JJ1886
Chen *et al.* 2006	Escherichia coli UTI89
Welch *et al.* 2002	Escherichia coli CFT073
Oshima *et al.* 2008	Escherichia coli SE11
Avasthi *et al.* 2011	Escherichia coli NA114
Petty *et al.* 2014	ENI Run Accession IDs:<br>ERR161327, ERR161319, ERR161312, ERR161324, ERR161235, ERR161245, ERR161302, ERR161300, ERR161236, ERR161257, ERR161283, ERR161305, ERR161291, ERR161267, ERR161268, ERR161242, ERR161243, ERR161318, ERR161244, ERR161299, ERR161277, ERR161301, ERR161241, ERR161315, ERR161308, ERR161323, ERR161325, ERR161304, ERR161278, ERR161266, ERR161303, ERR458470, ERR161282, ERR161281, ERR161320, ERR161328, ERR161285, ERR161252, ERR161261, ERR161260, ERR161262, ERR161288, ERR161274, ERR161272, ERR161284, ERR161294, ERR161240, ERR161286, ERR161289, ERR161295, ERR161293, ERR161259, ERR161276, ERR161264, ERR161250, ERR161246, ERR161249, ERR161254, ERR161290, ERR161251, ERR161297, ERR161248, ERR161237, ERR161239, ERR161317, ERR161322, ERR161321, ERR161296, ERR161287, ERR161292, ERR458473, ERR458472, ERR458471, ERR161256, ERR161234, ERR161238, ERR161314, ERR161255, ERR161280, ERR161279, ERR161313, ERR161258, ERR161310, ERR161326, ERR161306, ERR161329, ERR161307, ERR161311, ERR161263, ERR161273, ERR161265, ERR161275, ERR161253, ERR161316, ERR161309, ERR161298, ERR161270, ERR161271
Salipante *et al*. 2014	NCBI Biosample IDs:<br>SAMN02802029, SAMN02801911, SAMN02802151, SAMN02801841, SAMN02801913, SAMN02801926, SAMN02802184, SAMN02802164, SAMN02802014, SAMN02802016, SAMN02801851, SAMN02802002, SAMN02801996, SAMN02802006, SAMN02802128, SAMN02802023, SAMN02801880, SAMN02802191, SAMN02802041, SAMN02801842, SAMN02802131, SAMN02801965, SAMN02801853, SAMN02802194, SAMN02801973, SAMN02802150, SAMN02801957, SAMN02802056, SAMN02802059, SAMN02801892, SAMN02801823, SAMN02801881, SAMN02801919, SAMN02802081, SAMN02802139, SAMN02802174, SAMN02802169, SAMN02802060, SAMN02802193, SAMN02802068, SAMN02802167, SAMN02801952, SAMN02801825, SAMN02801921, SAMN02801994, SAMN02802109, SAMN02801940, SAMN02802172, SAMN02801924, SAMN02802123, SAMN02801949, SAMN02802030, SAMN02801985, SAMN02801954, SAMN02801945, SAMN02801992, SAMN02802143, SAMN02802074, SAMN02802101, SAMN02802136, SAMN02801969, SAMN02802099, SAMN02802026, SAMN02801968, SAMN02802117, SAMN02801875, SAMN02802035, SAMN02801874, SAMN02801854, SAMN02801894, SAMN02801933, SAMN02802085, SAMN02802069, SAMN02801902, SAMN02801840, SAMN02802022, SAMN02801844, SAMN02801974, SAMN02802115, SAMN02802003, SAMN02802158, SAMN02801925, SAMN02802155, SAMN02801828, SAMN02801903, SAMN02801832, SAMN02801977, SAMN02801814, SAMN02802048, SAMN02801914, SAMN02801852, SAMN02801989, SAMN02801971, SAMN02801951, SAMN02802049, SAMN02802093, SAMN02801888, SAMN02801999, SAMN02802087, SAMN02802052, SAMN02801923, SAMN02802053, SAMN02802079, SAMN02801998, SAMN02801885, SAMN02802170, SAMN02802153, SAMN02802083, SAMN02801846, SAMN02801943, SAMN02802073, SAMN02801818, SAMN02801944, SAMN02801972, SAMN02801816, SAMN02802057, SAMN02802028, SAMN02801982, SAMN02801915, SAMN02801950, SAMN02802088, SAMN02801928, SAMN02802061, SAMN02801815, SAMN02802113, SAMN02802044, SAMN02802076, SAMN02801906, SAMN02801895, SAMN02801947, SAMN02802005, SAMN02802175, SAMN02802182, SAMN02802050, SAMN02801879, SAMN02801834, SAMN02801831, SAMN02801824, SAMN02801876, SAMN02802015, SAMN02801822, SAMN02802034, SAMN02801978, SAMN02801896, SAMN02801838, SAMN02801993, SAMN02802183, SAMN02802043, SAMN02802103, SAMN02801839, SAMN02801889, SAMN02802180, SAMN02802186, SAMN02802146, SAMN02801819, SAMN02801865, SAMN02801866, SAMN02802004, SAMN02801833, SAMN02802063, SAMN02801890, SAMN02801897, SAMN02802129, SAMN02801941, SAMN02801958, SAMN02801935, SAMN02802154, SAMN02801937, SAMN02801916, SAMN02801953, SAMN02801948, SAMN02801961, SAMN02801988, SAMN02802077, SAMN02801936, SAMN02801868, SAMN02801843, SAMN02801817, SAMN02801938, SAMN02802012, SAMN02801882, SAMN02801862, SAMN02802116, SAMN02802084, SAMN02801826, SAMN02802187, SAMN02802062, SAMN02801820, SAMN02802163, SAMN02801907, SAMN02801821, SAMN02801918, SAMN02801856, SAMN02802110, SAMN02801827, SAMN02801927, SAMN02802054, SAMN02801883, SAMN02801932, SAMN02801990, SAMN02802185, SAMN02801910, SAMN02801847, SAMN02801864, SAMN02801917, SAMN02802100, SAMN02802171, SAMN02802142, SAMN02802024, SAMN02801886, SAMN02801964, SAMN02801829, SAMN02802121, SAMN02801859, SAMN02802173, SAMN02801812, SAMN02801873, SAMN02802168, SAMN02801878, SAMN02802082, SAMN02802033, SAMN02801970, SAMN02801900, SAMN02801899, SAMN02801858, SAMN02801861, SAMN02801871, SAMN02801891, SAMN02801960, SAMN02801922, SAMN02801963, SAMN02801939, SAMN02801930, SAMN02801836, SAMN02802130, SAMN02801884, SAMN02802148, SAMN02801966, SAMN02801835, SAMN02802104, SAMN02801850, SAMN02801869, SAMN02802162, SAMN02801857, SAMN02802178, SAMN02801959, SAMN02801946, SAMN02802177, SAMN02802114, SAMN02802119, SAMN02801962, SAMN02802080, SAMN02802120, SAMN02801904, SAMN02802179, SAMN02801901, SAMN02801845, SAMN02801860, SAMN02801905, SAMN02801867, SAMN02802176, SAMN02802195, SAMN02801837, SAMN02801929, SAMN02802132, SAMN02802159, SAMN02801813, SAMN02801987, SAMN02801855, SAMN02802096, SAMN02801830, SAMN02801956, SAMN02801872, SAMN02802147, SAMN02802021, SAMN02801909, SAMN02802118, SAMN02801955, SAMN02802126, SAMN02801848, SAMN02802189, SAMN02801870, SAMN02801849, SAMN02802064, SAMN02801893, SAMN02802157, SAMN02802051

Supplementary Table S5: Distribution of unique accessory genes identified through pan-genome analysis responsible for hierarchical clustering topology.

Cluster C1	Cluster C2	Cluster C3	ST-131	ST-219
yaaJ, azoR, bcsA, bhsA, caeB, clpP1, ddpA, dgoD, dicA, dicC, dinD, dosC, epsE, fhuE, fliC, fruB, frvR, fucR, ghrB, gldA, hyaA, hyfR, insG, insN-1, intA, intQ, intZ, leuB, lldP, lomR, mak, mbeA, mbeC, mceA, mceB, mec, metB, metN, mshA, mtlA, mtlD, namA, nmpC, nohA, ompR, oppA, parD1, potC, preA, relB, relE, rffE, rna, rpe, rtcB, rzpQ, sadB, slrP, slt, sohB, ssb, stfE, tap, tfaD, tfaR, tfaX, ucpA, yafX, yajI, ydcR, yddK, ydfE, ydfH, ydfV, ydiB, ydjE, yeaX, yedK, yfaL, yfaV, yfcG, yfdS, yfdT, yffO, ygeF, yjcS, yncG, ynfO, yniC	*ysaB, cnu, raiA, ldrD, ghrB, rplY, mokC, acpP, pliG, yqaE, bglG*	*renD, arpA, ybcN, ybcK*	*insC-1, dsdC, papB, uidB, yjbH, yeeT, yfjQ, waaK, dpnM, yejO, dppB, virF, paaY, kduD, tarF, insI-2, wbbJ, yfaX, rzpD, lomR, hlyC, insD, malY, rusA, axeA1, yphA, uidR, entF, intA, ygfI, ygfI, yicI, yjjJ, hsdR, rfaZ, rfaY, bshA, rfaS, rfaP, yadD, fimA, exuT, yfbK, yfcS, yeeJ, tabA, yiaM, yiaN, immR, yejO, gatC, rfbC, mntH, crt, carA, pnuC, nadR, sucA, sucB, lpd, succC, sucD, ldh, dctD, oxyR, cidA, yohK, oppF, dppD, hxlB, ptsG, tktA, ybbH, yqiG, uxuR, ydfI, yjjN, mprA, yjmC, siaQ, ygiZ, yfbS, tabA, ygdQ, yagE, adh1, pdxA, glcR, lutR, yjmD, uxaA, suyB, yiaO, siaM, fixX, sfsB, fimD, insB, tsh, espC, arsC, gpFI, sppA, tfaD, ybcO, ydaT, dicA, leuO, nimR, emrK, emrB, mntB, wrbA, fabG, rbsC, rbsA, glpK, fas2, dxs, yeeW, yfjI, intA, ykgN, xerC, ykfG, intD, cca, cadC, wcaA, rfaE, wbbK, wbbL, aes, rzpD, borD, tonB, fabH, mcrB, mtnK, rzpD, cysH, glmU, tnpA, yagE, pdxA2, sauU, ulaA, ymfN, higB-1, resE, yedW, yedY, hlyA, hlyD, toxA, insF-1, ompW, iucA, iucB, iucC, iucD, iutA, emrE, pduL, ccmK, cutD, cutC, adhE, ccmL, pduA, eutM, mtrR, cdiA, cdiI, dcm, papE, prsF, papG, pagN, yjdJ, yahA, yiaD, yfjJ, mleN, cirA, nanS, lutR, fliD, crfC, fhuA, btuF, hmuU, fepC, htrE, yadV, Hmo, nemR, ydfX, dicC, dicA, ydfA, dicB, ydfD, nanM, nanC, axeA1, nanT, nanK, nanA, ompF, alpA, traC, dicC, dicA, epsL, kpsM, malK, bspRIM, stfR*	*upaG, fimI, intB, yagA, dmsA, dmsB, ynfH, yfcO, yfbL, smc, yedL, hsdM, pcrA, lhr, hepA, rhlE, pglA, galE, rffD, pikAIV, gcvT, fabZ, acpS, shlB, cdiA2, fimA, yadV, yqeI, yqeJ, rtn, yidJ, ulaB, wcaB, yadV, yicJ, yabP, yabP, ymfR, stfE, yedK, ydfG, ycjZ, ycjY, nepI, yaiU, marA, afuC, afuB, potD, glpT, uhpB, fimZ, Smc, ygeG, ygcG, yfiN, pstS1, lhr, ydhY, insB-4, ompN, nanM, ytfI, ipaH3, yahH, ypjB*

Supplementary Table S6: Distribution of PAI- CFT073 and 536 associated genes in the genomes of the 11 sequenced strains.

				Phylogroup A				Phylogroup B1		Phylogroup B2			Phylogroup D
				ST-361	ST-410	ST4204		ST-448	Unknown ST	ST-131			ST-59
**Pathogenicity Island**	**Genbank Accesion**	**Gene**	**Function**	**NGCE94**	**NGCE33**	**NGE22**	**NGE4**	**NGCE100**	**NGE5**	**NGE7**	**NGE16**	**NGE9**	**NGE3**
PAI ICFT073	AF003742	*licT*	Alpha hemolysin, P fimbriae, aerobactin	N	N	N	N	N	N	Y	Y	N	Y
		malX		N	N	N	N	N	N	Y	Y	N	Y
	AF081283	*dadX*		Y	Y	Y	Y	Y	Y	Y	Y	Y	Y
		cvrA		Y	Y	Y	Y	Y	Y	Y	Y	N	Y
		ycgR		Y	Y	Y	Y	Y	Y	Y	Y	N	Y
		emtA		Y	Y	Y	Y	Y	Y	Y	Y	N	Y
		ldcA		Y	Y	Y	Y	Y	Y	Y	Y	N	Y
		ubiE		N	N	N	N	N	N	Y	Y	N	Y
	AF081284	*hlyD*		N	N	N	N	N	N	Y	Y	N	N
PAI IICFT073	AF447814	*cbeA*	P fimbriae, iron regulation	Y	Y	Y	Y	Y	N	Y	Y	N	Y
		cadB		Y	Y	Y	Y	Y	Y	Y	Y	Y	Y
		cadC		Y	Y	Y	Y	Y	Y	Y	Y	Y	Y
		cadA		Y	Y	Y	Y	Y	Y	Y	Y	Y	Y
		klcA		N	N	N	N	Y	Y	Y	N	N	Y
		cbtA		Y	N	N	N	N	N	N	N	N	N
		der		Y	N	N	N	Y	N	Y	Y	N	N
		papK		Y	N	N	N	N	N	Y	Y	N	Y
		papB		Y	N	N	N	N	N	N	Y	N	N
		papD		Y	N	N	N	N	N	Y	Y	N	N
		papH		Y	N	N	N	N	N	Y	Y	N	N
		papC		Y	N	N	N	N	N	Y	Y	N	N
		prsF		N	N	N	N	N	N	Y	Y	N	N
		papG		N	N	N	N	N	N	Y	Y	N	N
		crfC		N	N	N	N	Y	N	Y	Y	N	N
		hmuU		N	N	N	N	N	N	Y	Y	N	N
		fepC		N	N	N	N	N	N	Y	Y	N	N
		fatA		N	N	N	N	N	N	Y	Y	N	N
		fcuA		N	N	N	N	N	N	N	N	N	N
		cadA		N	N	N	N	N	N	N	N	N	N
PAI I536	AJ488511	*klcA*	Alpha hemolysin, fimbriae, adhesins	N	N	N	N	Y	Y	Y	N	N	Y
		cbtA		Y	N	N	N	N	N	N	N	N	N
		hlyD		N	N	N	N	N	N	Y	Y	N	N
		hlyA		N	N	N	N	N	N	Y	Y	N	N
		hlyC		N	N	N	N	N	N	Y	Y	N	N
		hlyB		N	N	N	N	N	N	Y	Y	N	N
		ybdM		N	N	N	N	N	N	Y	Y	N	Y
		cysH		N	N	N	N	N	N	Y	Y	N	N
		htrE		N	N	N	N	N	N	Y	Y	N	N
		yadV		N	N	N	N	N	N	Y	Y	N	N
		yphA		N	N	N	N	N	N	Y	Y	N	N
		hmo		N	N	N	N	N	N	Y	Y	N	N
		srpR		N	N	N	N	N	N	Y	Y	N	N
		cfaD		N	N	N	N	N	N	N	N	N	N
		fimA		N	N	N	N	N	N	N	N	N	N
		elfC		N	N	N	N	N	N	N	N	N	N
		mntB		N	N	N	N	N	N	N	N	N	N
		mntA		N	N	N	N	N	N	N	N	N	N
		cfaD		N	N	N	N	N	N	N	N	N	N

Supplementary Table S6 continued:

PAI II536	AJ494981	*cbeA*	Alpha hemolysin, P fimbriae, hekadhesin, hemagglutinin like adhesins	Y	Y	Y	Y	Y	N	Y	Y	N	Y
		klcA		N	N	N	N	Y	Y	Y	N	N	Y
		opgE		Y	N	N	N	Y	N	Y	Y	N	Y
		der		Y	N	N	N	Y	N	Y	Y	N	N
		dsdC		Y	N	Y	Y	N	N	Y	Y	N	Y
		papK		Y	N	N	N	N	N	Y	Y	N	Y
		papB		Y	N	N	N	N	N	N	Y	N	N
		papD		Y	N	N	N	N	N	Y	Y	N	N
		dsdX		Y	N	Y	Y	N	N	N	Y	N	Y
		papH		Y	N	N	N	N	N	Y	Y	N	N
		papC		Y	N	N	N	N	N	Y	Y	N	N
		hlyD		N	N	N	N	N	N	Y	Y	N	N
		prsF		N	N	N	N	N	N	Y	Y	N	N
		intA		N	N	N	N	Y	Y	Y	Y	N	N
		yciC		N	N	N	N	N	N	Y	Y	N	Y
		recD2		Y	N	N	N	N	N	Y	Y	N	N
		hlyA		N	N	N	N	N	N	Y	Y	N	N
		hlyC		N	N	N	N	N	N	Y	Y	N	N
		hlyB		N	N	N	N	N	N	Y	Y	N	N
		yedZ1		N	N	N	N	N	N	Y	Y	N	N
		cdiA		N	N	N	N	N	N	Y	Y	N	N
		pagN		N	N	N	N	N	N	Y	Y	N	N
		phoP		N	N	N	N	N	N	Y	Y	N	N
		ydiO		N	N	N	N	N	N	Y	Y	N	N
		cdiI		N	N	N	N	N	N	Y	Y	N	N
		shlB		N	N	N	N	N	N	Y	Y	N	N
		emrE		N	N	N	N	N	N	Y	Y	N	N
		pduL		N	N	N	N	N	N	Y	Y	N	N
		ccmK		N	N	N	N	N	N	Y	Y	N	N
		cutD		N	N	N	N	N	N	Y	Y	N	N
		cutC		N	N	N	N	N	N	Y	Y	N	N
		adhE		N	N	N	N	N	N	Y	Y	N	N
		ccmL		N	N	N	N	N	N	Y	Y	N	N
		pduA		N	N	N	N	N	N	Y	Y	N	N
		mtrR		N	N	N	N	N	N	Y	Y	N	N
		patB		N	N	N	N	N	N	Y	Y	N	N
		yedY1		N	N	N	N	N	N	Y	Y	N	N
		mleN		N	N	N	N	N	N	Y	Y	N	N
		resE		N	N	N	N	N	N	N	Y	N	N
		dsdA		N	N	N	N	N	N	Y	N	N	N
		nhaC		N	N	N	N	N	N	N	N	N	N
PAI III536	X16664	*yciC*	S fimbriae, iron siderophore system, Sap adhesin, TSH like hemoglobin protease, HmuR like heme receptor	N	N	N	N	N	N	Y	Y	N	Y
PAI IV536	AF135406	*mtfA*	Siderophore synthesis, iron uptake	Y	Y	Y	Y	Y	Y	Y	Y	Y	Y
		intA		N	N	Y	Y	Y	N	Y	Y	N	Y
		yodB		Y	Y	N	N	N	Y	N	N	Y	N
	AF136296	*fyuA*		N	N	Y	Y	N	N	Y	Y	N	Y
PAI V536	AJ617685	*intS*	K15 Capsule	Y	N	Y	Y	N	N	Y	N	N	Y
		cbeA		Y	Y	Y	Y	Y	N	Y	Y	N	Y
		gspK		Y	Y	N	N	Y	Y	Y	Y	Y	N
		acpP		Y	Y	N	N	Y	Y	Y	Y	Y	N
		yghQ		Y	Y	N	N	Y	Y	Y	Y	Y	N
		glcA		Y	Y	N	N	Y	Y	Y	Y	Y	N
		glcB		Y	Y	N	N	Y	Y	Y	Y	Y	N
		lutA		Y	Y	N	N	Y	Y	Y	Y	Y	N
		glcC		Y	Y	N	N	Y	Y	Y	Y	Y	N
		bioF		Y	Y	N	N	Y	Y	Y	Y	Y	N
		oleD		Y	Y	N	N	Y	Y	Y	Y	Y	N
		lptG		Y	Y	N	N	Y	Y	Y	Y	Y	N
		lptF		Y	Y	N	N	Y	Y	Y	Y	Y	N
		ytfJ		Y	Y	N	N	Y	Y	Y	Y	Y	N
		cbtA		Y	N	N	N	N	N	N	N	N	N
		rbsK		Y	Y	N	N	N	N	Y	Y	N	N
		xcpW		Y	N	N	N	Y	Y	N	N	Y	N
		epsF		Y	N	N	N	Y	Y	N	N	Y	N
		epsE		Y	N	N	N	Y	Y	N	N	Y	N
		fucP		Y	Y	N	N	N	N	Y	Y	N	N
		epsH		Y	N	N	N	Y	Y	N	N	Y	N
		epsG		Y	N	N	N	Y	Y	N	N	Y	N
		epsD		Y	N	N	N	Y	Y	N	N	Y	N
		xcpV		Y	N	N	N	Y	Y	N	N	Y	N
		epsC		Y	N	N	N	Y	Y	N	N	Y	N
		pppA		Y	N	N	N	Y	Y	N	N	Y	N
		kpsF		N	N	N	N	N	N	Y	Y	N	Y
		flu		Y	N	Y	N	N	N	Y	N	N	N
		kpsU		N	N	N	N	N	N	Y	Y	N	Y
		dctD		N	N	Y	Y	N	N	N	N	N	N
		pdeL		N	N	Y	Y	N	N	N	N	N	N
		papF		N	N	Y	Y	N	N	N	N	N	N
		papD		N	N	Y	Y	N	N	N	N	N	N
		papC		N	N	Y	Y	N	N	N	N	N	N
		pgtC		N	N	N	N	N	N	N	N	N	N
		pgtB		N	N	N	N	N	N	N	N	N	N
		pgtP		N	N	N	N	N	N	N	N	N	N
